# Supplementary material for: LDL receptor-related protein 5 selectively transports unesterified polyunsaturated fatty acids to intracellular compartments
Source: Nat Commun. 2024 Apr 9;15:3068. doi: 10.1038/s41467-024-47262-z (PMC11004178; doi:10.1038/s41467-024-47262-z)
Supplement: Supplementary file 1 — Supplementary Information [file 41467_2024_47262_MOESM1_ESM.pdf]

SUPPLEMENTARY INFORMATION

LDL Receptor-Related Protein 5 Selectively Transports Unesterified Polyunsaturated Fatty Acids to Intracellular Compartments

Wenwen Tang[1,2*†], Yi Luan[1,2*], Qianying Yuan[1,2*], Ao Li[1,2], Song Chen[2], Stanley Menacherry[3], Lawrence Young[4,5,6], Dianqing Wu[1,2†]

[1]Vascular Biology and Therapeutic Program, Yale University School of Medicine, New Haven CT 06520, USA.

[2]Department of Pharmacology, Yale University School of Medicine, New Haven CT 06520, USA.

[3]Gateway Community College, New Haven, CT 06510, USA

[4]Cardiovascular Research Center, Yale University School of Medicine, New Haven CT 06520, USA.

[5]Department of Internal Medicine (Cardiovascular Medicine), Yale University School of Medicine, New Haven CT 06520, USA.

[6]Department of Cellular and Molecular Physiology, Yale University School of Medicine, New Haven CT 06520, USA

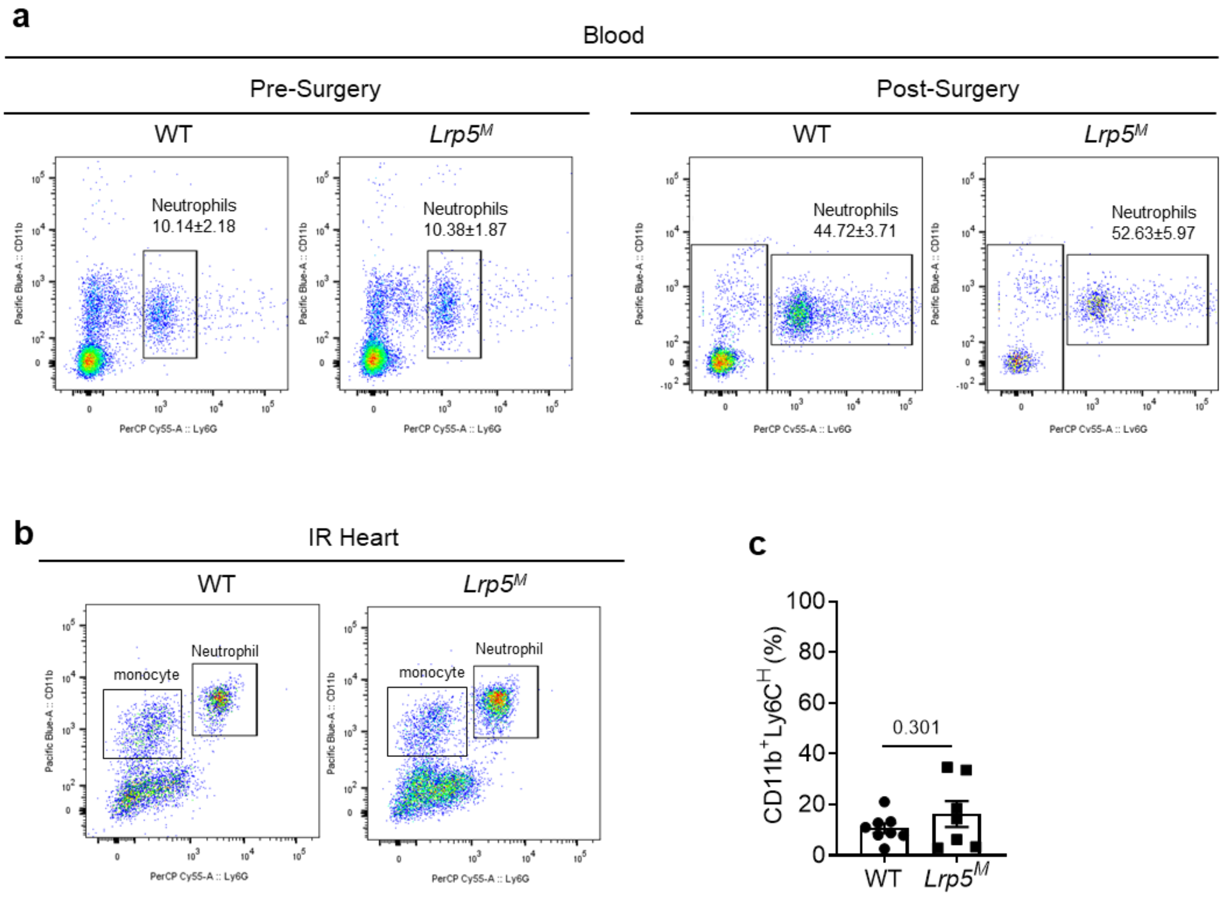

Supplementary Figure 1. Myeloid-specific LRP5, but not LRP6, KO, leads to increased myocardial ischemia reperfusion injury and NET formation. a Flowcytometry analyses of blood neutrophils (n=6 in pre-surgery WT and n=5 in pre-surgery *Lrp5*^{M}; n=10 in post-surgery WT and n=8 in post-surgery *Lrp5*^{M}). Data are presented as mean±sem. No significance between WT and KO mice (Student's t-test, Two-tailed, unpaired). **b** Representative flowcytometry charts for Fig. 1d. **c** Monocyte presence in injured hearts determined by flowcytometry (n=8 in WT and n=7 in *Lrp5*^{M}). Data are presented as mean±sem with p values (Student's t-test, Two-tailed, unpaired).

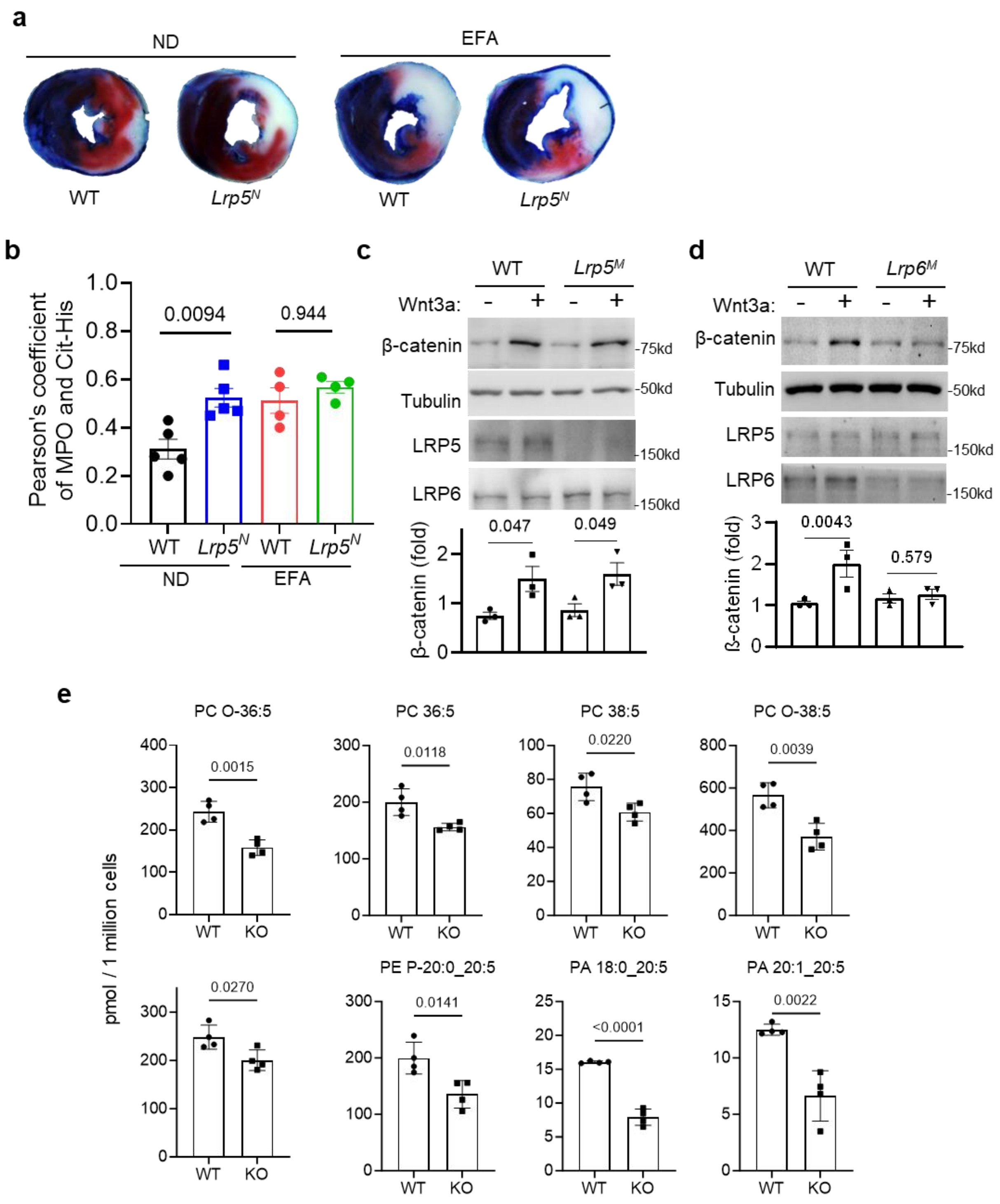
a
ND
EFA
WT
Lrp5^N
WT
Lrp5^N
b
Pearson's coefficient of MPO and Cit-His
0.0094
0.944
WT
Lrp5^N
WT
Lrp5^N
ND
EFA
c
WT
Lrp5^M
Wnt3a:
β-catenin
Tubulin
LRP5
LRP6
-75kd
-50kd
-150kd
-150kd
β-catenin (fold)
0.047
0.049
d
WT
Lrp6^M
Wnt3a:
β-catenin
Tubulin
LRP5
LRP6
-75kd
-50kd
-150kd
-150kd
β-catenin (fold)
0.0043
0.579
e
pmol / 1 million cells
PC O-36:5
0.0015
PC 36:5
0.0118
PC 38:5
0.0220
PC O-38:5
0.0039
0.0270
PE P-20:0_20:5
0.0141
PA 18:0_20:5
<0.0001
PA 20:1_20:5
0.0022
WT
KO

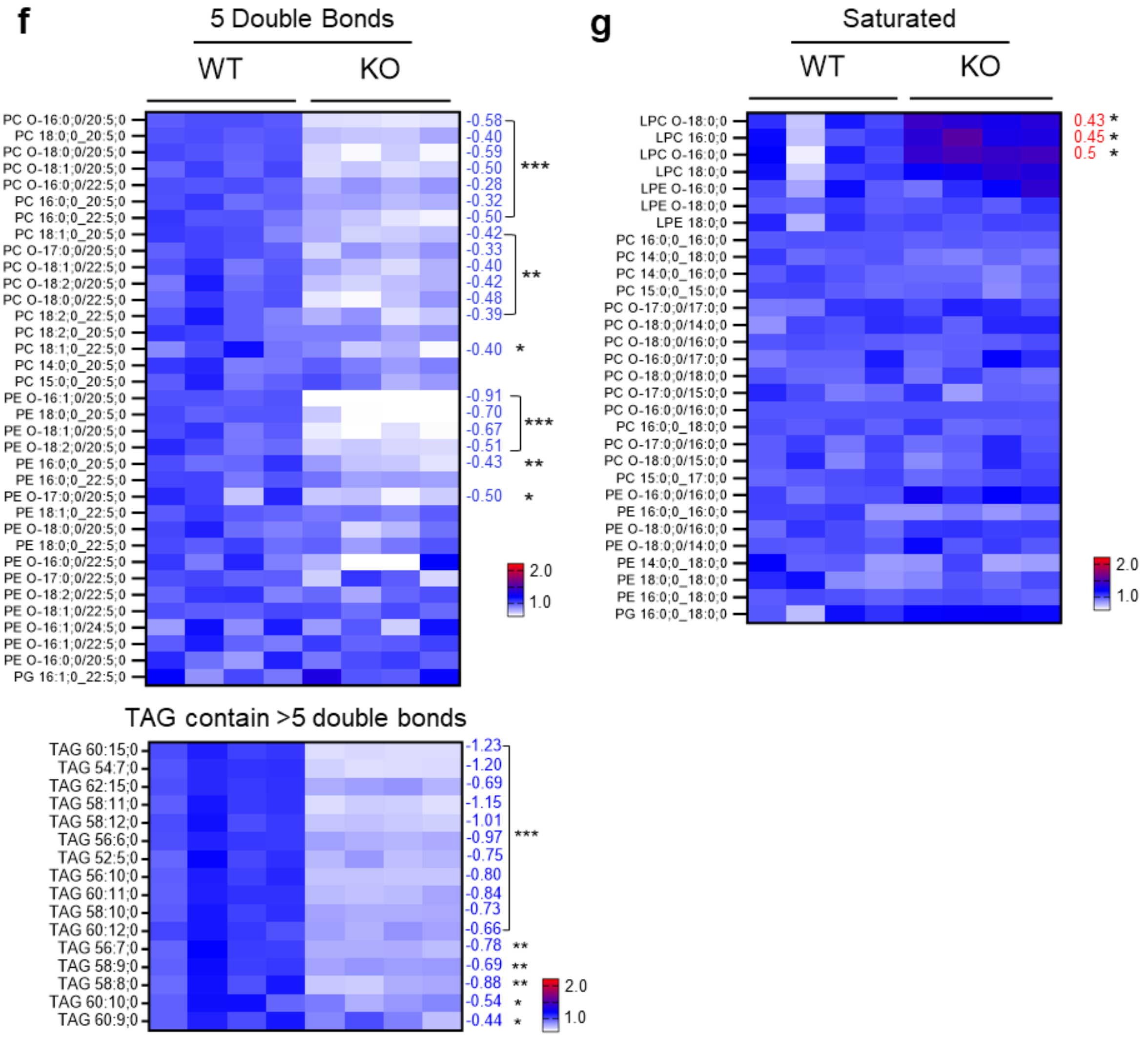
f
5 Double Bonds
WT
KO
TAG contain >5 double bonds
g
Saturated
WT
KO
2.0
1.0

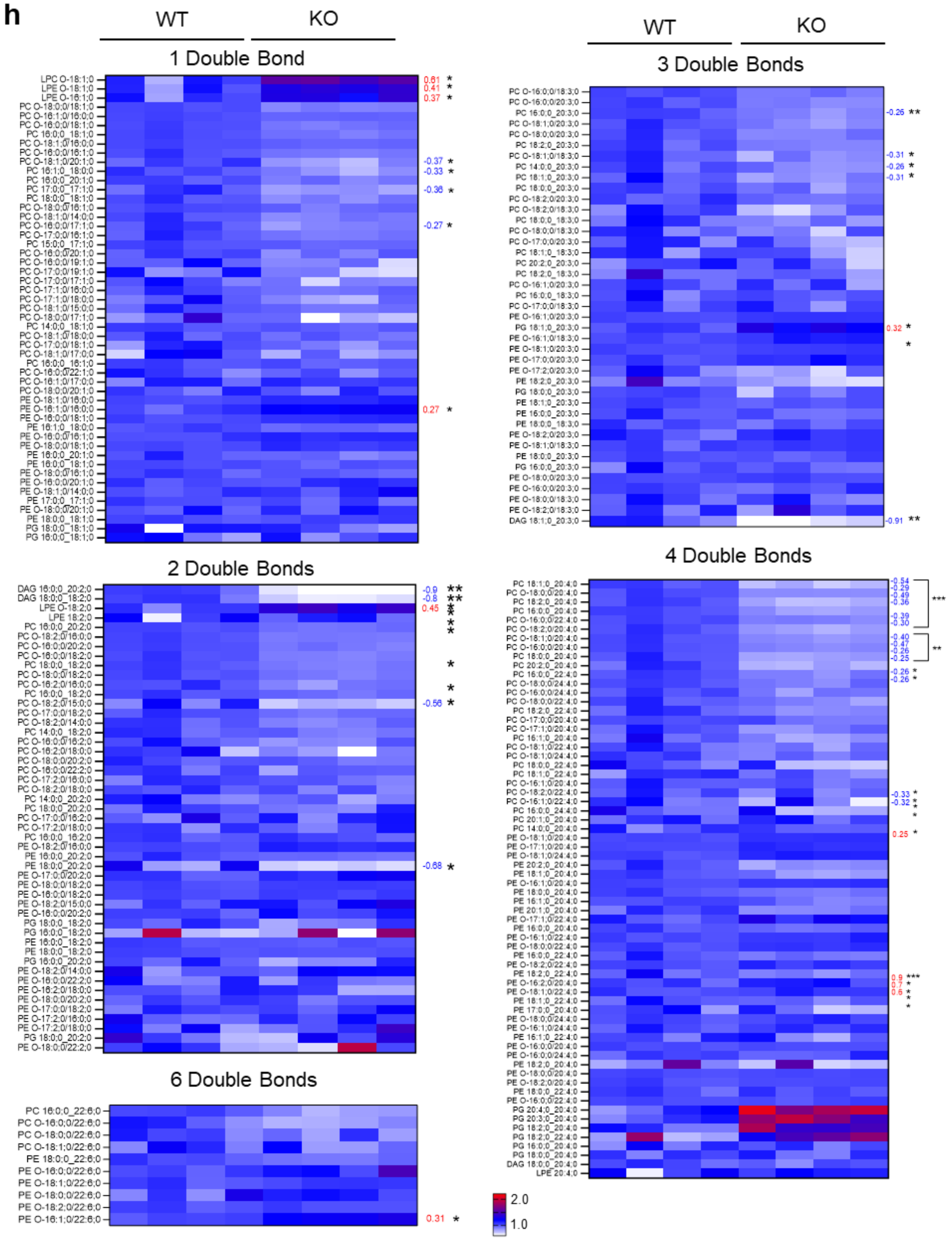
h
WT
KO
1 Double Bond
2 Double Bonds
6 Double Bonds
3 Double Bonds
4 Double Bonds
2.0
1.0

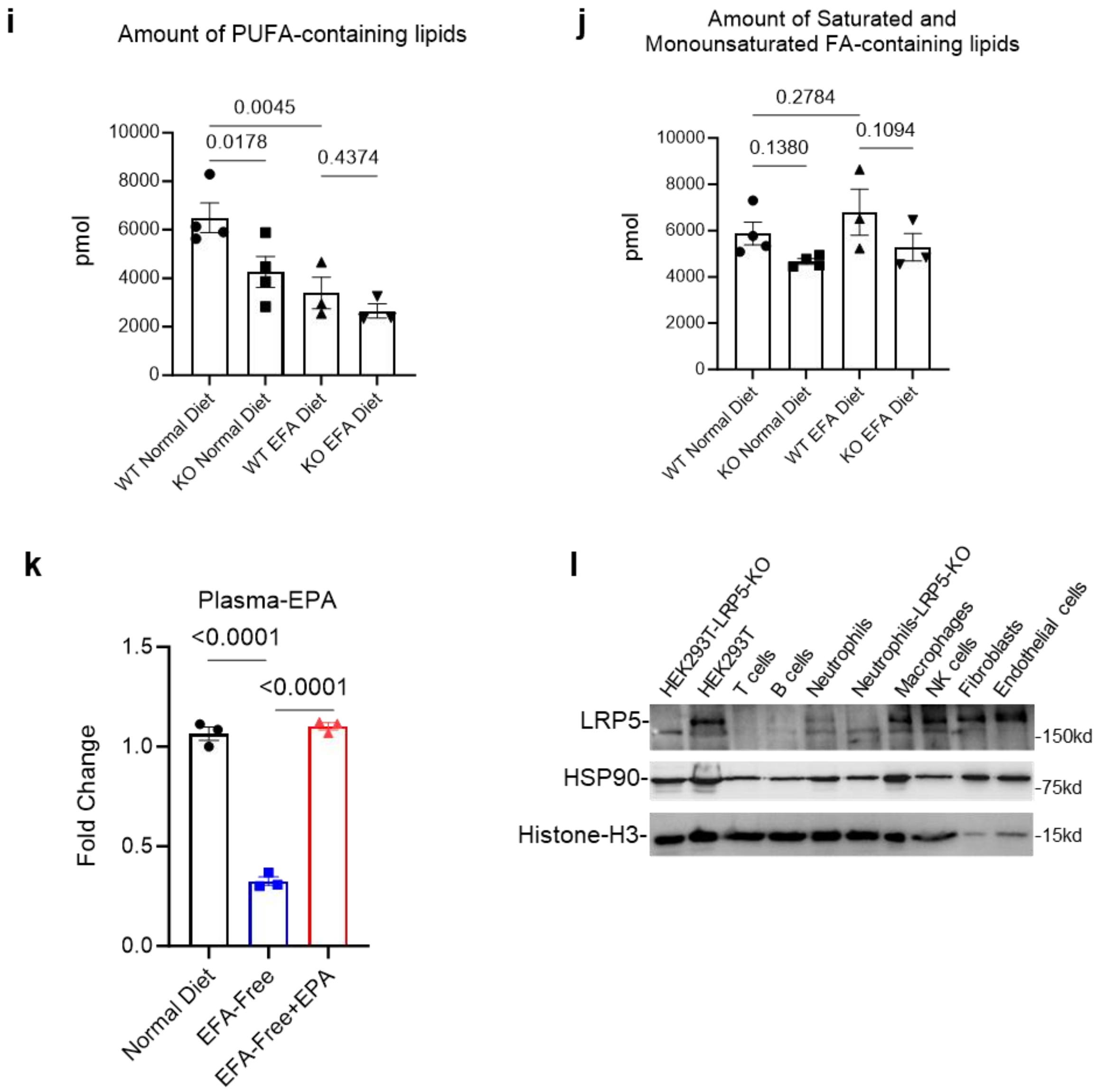

Supplementary Figure 2. LRP5-deficiency does not affect Wnt-β-catenin signaling, but reduces PUFA accretion in neutrophils. a Representative heart section images stained with triphenyl tetrazolium chloride from mice subjected to myocardial ischemia reperfusion injury for Fig. 2a. **b** Pearson's coefficients of co-localization of MPO and citrullinated histone (c-His) in the injured heart sections. Each datum point is one mouse (n=5 per group for ND treatment and n-4 per group for EFA treatment). Data are presented as mean±sem with p values (One-way ANOVA) **c, d** Bone marrow neutrophils were isolated from *Lrp5*^{M} (**c**) or *Lrp6*^{M} (**d**) and subjected to Wnt3A stimulation (50 ng/ml, 2 hours). **e** Targeted

LC-MS analysis of PC, PE and PS species in neutrophils that were isolated from neutrophil-specific LRP5 KO or WT mice. Data in **b-e** are presented as mean±sem with p values (Student's t-test, Two-tailed, unpaired). **f, g** Heatmaps of relative amounts of all of the phospholipids with one of fatty acid chains containing 5 double-bonds, triglycerides containing more than 5 double bonds (**f**), and phospholipids containing only saturated fatty acids (**g**) that were identified by the lipidomic analysis. **h** Heatmap of all glycerolipid species identified by the lipidomic analysis that contain fatty acids with 1 double-bond, 2 double-bonds, 3 double-bonds, 4 double-bonds, 6 double bonds. Data in **f-h** are presented as Log_2fold changes of the content in LRP5 KO neutrophils compared to that in WT neutrophils. Significant reductions and increases (Log_2fold>0.25 or <-0.25; p<0.05, Student's t-test, Two-tailed, unpaired) are shown with the Log_2fold change values at the right side in blue or red, respectively. (* p<0.05, ** p<0.01, *** p<0.001). The complete dataset is shown in Supplementary Data 1. **i, j** Lipidomic analysis of PUFA-containing lipids (**i**) or saturated and monounsaturated fatty acids containing lipids (**j**) in neutrophils from WT or LRP5 KO mice on normal diet or EFA-Free diet. Data are presented as mean±sem with p values (One-way ANOVA). **k** Targeted LC-MS analysis of EPA level in the plasma of the mice on normal diet, EFA-free diet or refed with EPA after EFA-free diet. Data are presented as mean±sem with p values (One-way ANOVA). **l** Western blot of LRP5 protein expression in different cell types.

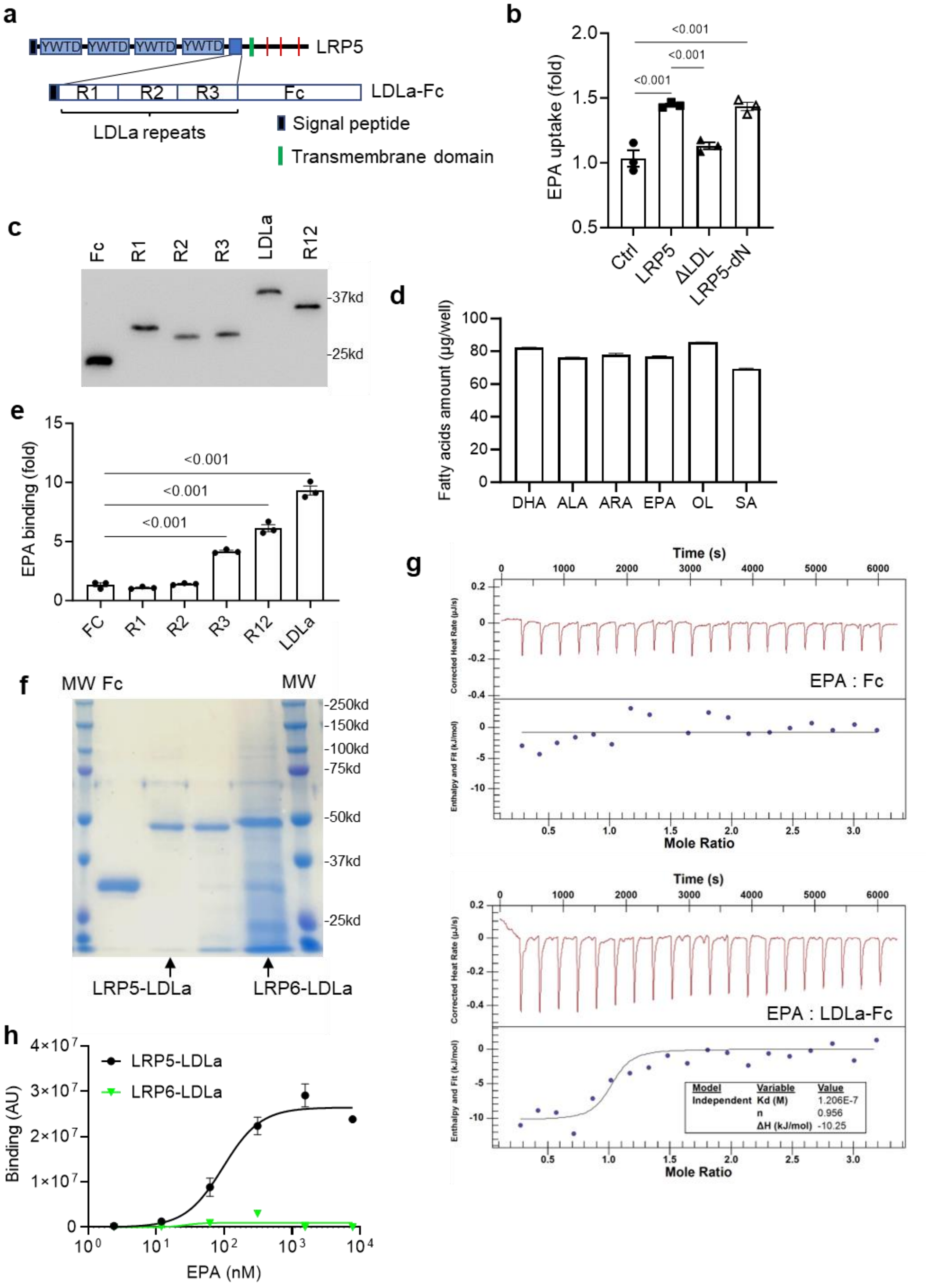

Supplementary Figure 3. LRP5 LDLa repeats bind to PUFAs. a Schematic representation of the full-length LRP5 protein and LRP5 LDLa-Fc fusion protein. **b** LRP5 KO HEK293T cells were transfected with a control vector (Ctr) or a plasmid expressing mouse LRP5, LRP5 without the LDLa repeats (ΔLDL) or LRP5 without N-terminal four YWTD repeats domains (LRP5-dN). The cells were incubated with ^{14}C-EPA with BSA. The uptake by the Ctr cells is taken as 1 (n=3 for each group). Data are presented as

mean±sem with p values (One-way ANOVA). **c** Western detection of LRP5 LDLa repeats fused with Fc in CMs from HEK293T cells. **d** The amounts of fatty acids that have been coated on the plate in the binding assays were determined by HPLC. **e** Binding of LRP5 LDLa repeats to fatty acids was determined by coating the plate with BSA or BSA plus a fatty acid, followed with incubation with conditioned medium from cells expressing Fc or LRP5 LDLa-Fc. The binding of Fc to BSA is set as 1 (n=3 for each group). Data are presented as mean±sem with p values (One-way ANOVA). **f** Coomassie blue stain of purified proteins. **g** Isothermal titration calorimetry assay was performed to determine the binding affinity between EPA and purified Fc (upper panel) or LDLa-Fc protein (bottom panel). **h** Binding of purified LRP5 LDLa repeats and LRP6-LDLa repeats fused with Fc to EPA. Binding to Fc was subtracted (n=3 for each group). Data are presented as mean±sem.

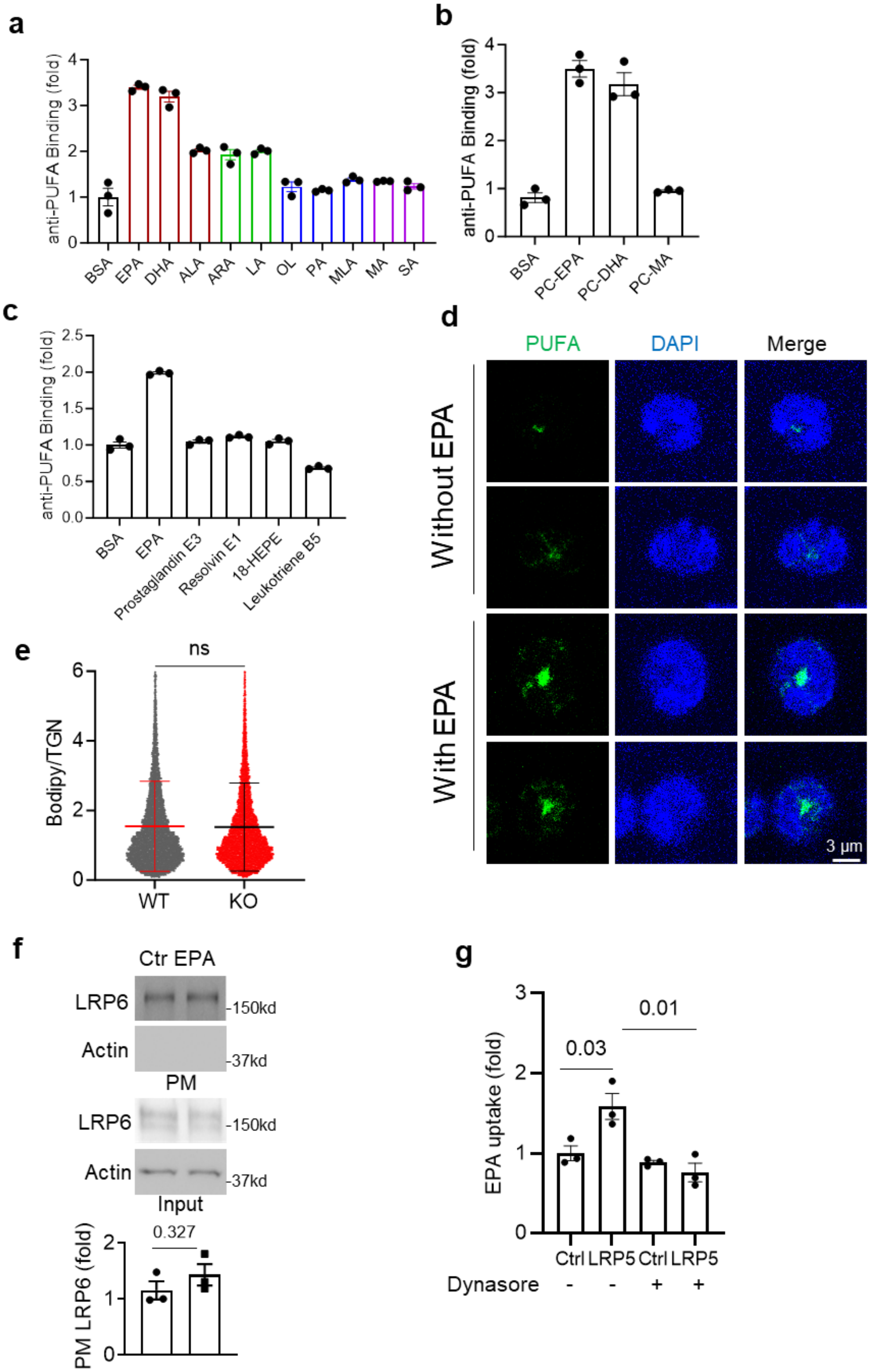

Supplementary Figure 4. Anti-PUFA specificity and LRP6 internalization. a The binding of anti-PUFA antibody to various fatty acids was determined by coating BSA or BSA carrying EPA, DHA, ALA, LA, OL (oleic acid), PA (palmitoleic acid), MLA (myristoleic acid), MA (myristic acid) or SA (steric acid) in wells of a 96-well plate and incubating the anti-PUFA antibody. Red bars represent n-3 PUFAs, green bars represent n-6 PUFAs, blue bars represent monounsaturated fatty acids and purple bars represent

saturated fatty acids (n=3 for each group). **b** The binding of anti-PUFA antibody to phosphocholine (PC) containing EPA, DHA, or myristic acid (MA) was determined as in a (n=3 for each group). **c** The binding of anti-PUFA antibody to EPA metabolites, including prostaglandin E3, resolving E1, 18-HEPE, and Leukotriene B5, was determined as in a (n=3 for each group). **d** Neutrophils from WT mice on the essential fatty acid-free diet and cultured with or without EPA (100 µM) for three hours were stained with the anti-PUFA and DAPI. **e** Quantification of Bodipy-palmitate fluorescence intensity with normalization of TGN staining intensity based on flowcytometry imaging analysis of neutrophils from WT and neutrophil-specific LRP5 KO mice on the essential fatty acid-free diet that were cultured with Bodipy-palmitate and stained with anti-TGN38 (n=8178 cells for WT and n=8916 cells for KO). **f** HEK293T cells were transfected with a LRP6 expression plasmid, and the internalization experiment was performed as in Fig. 5f. **g** LRP5 KO HEK293T cells were transfected with a control vector (Ctr) or a plasmid expressing mouse LRP5. The cells were treated with or without 80µM dynasore and incubated with ^{14}C-EPA with BSA. The uptake by the Ctr cells is taken as 1. Data in this figure are all presented as mean±sem with p values (Student's t-test, Two-tailed, unpaired for **f** and One-way ANOVA for **g**).

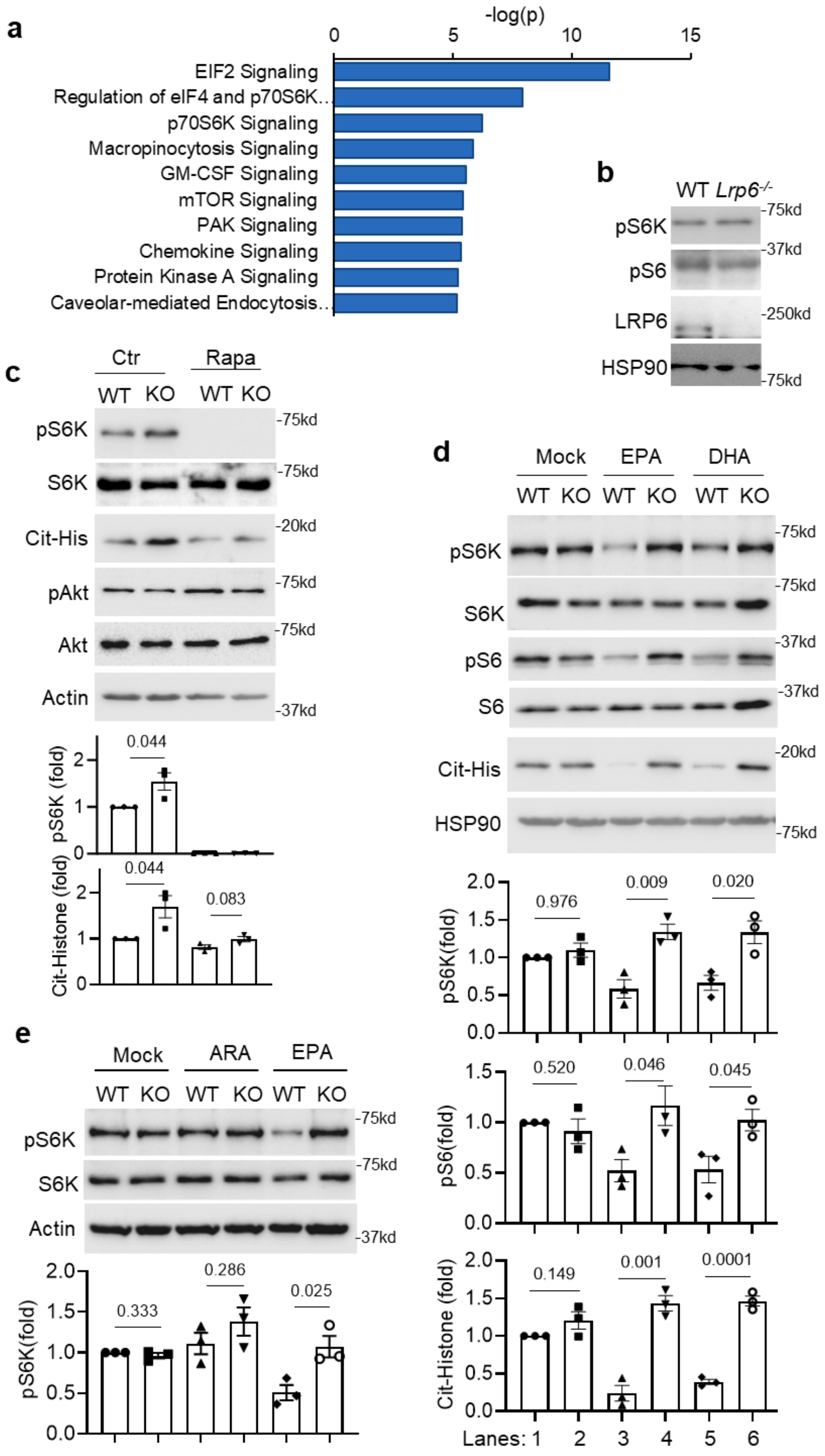

Supplementary Figure 5. LRP5-transported n-3 PUFA regulates mTORC1 signaling in neutrophils. **a** IPA pathway analysis of differentially expressed genes based on transcriptomic analysis of isolated bone marrow LRP5-null neutrophils in comparison with WT neutrophils. **b** Peritoneal neutrophils from WT

and myeloid-specific LRP6 KO (*Lrp6*$^{-/-}$) mice were analyzed by Western. **c** Peritoneal neutrophils from WT and neutrophil-specific LRP5 KO mice were cultured with10 ng/ml GM-CSF for four hours in the presence and absence of rapamycin (200 nM). Western qualification was normalized to actin (n=3 per group). Data are presented as mean±sem with p values (Student's t-test, Two-tailed, unpaired). **d, e** Neutrophils isolated from neutrophil-specific LRP5 KO or WT mice fed on the essential fatty acid-free diet were cultured with mock or 100 µM n-3 PUFAs as well as 10 ng/ml GM-CSF in the charcoal-filtered serum medium for three hours before being analyzed by Western blotting. Western qualification was normalized to HSP90 or actin. Data are presented as mean±sem with p values (Student's t-test, Two-tailed, unpaired).

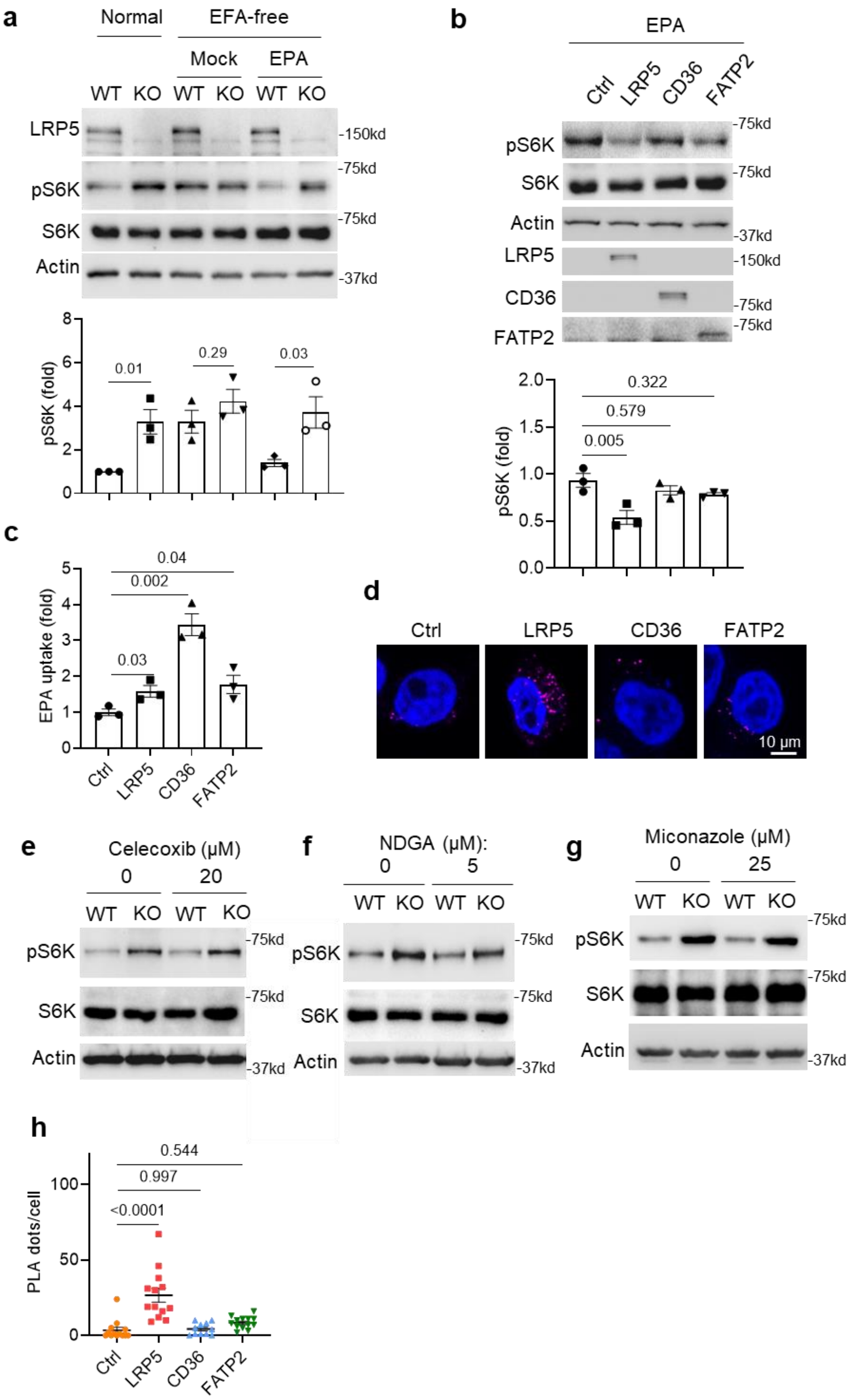

Supplementary Figure 6. LRP5-transported n-3 PUFA regulates mTORC1 signaling in HEK293T cells and COX2/lipoxygenase-mediated PUFA metabolites is not involved in LRP5-dependent mTORC1 regulation. a WT and LRP5 KO HEK293T cells were cultured in the normal or charcoal-filtered

serum overnight, followed by being cultured in the presence or absence of EPA for two hours before Western blotting analysis (n=3 per group). Data are presented as mean±sem with p values (Student's t-test, Two-tailed, unpaired). **b** LRP5 KO HEK293T cells transfected with the control plasmid (Ctrl), LRP5, CD36, or FATP2 were cultured in the charcoal-filtered serum overnight, followed by being cultured in the presence of EPA for two hours before western blotting analysis (n=3 per group). Data are presented as mean±sem with p values (One-way ANOVA). **c** LRP5 KO HEK293T cells were transfected with a control vector (Ctr), a plasmid expressing LRP5, CD36, or FATP2. EPA uptake was measured and analyzed as Supplementary Fig. 3b (n=3 per group). Data are presented as mean±sem with p values (Student's t-test, Two-tailed, unpaired). **d** LRP5 KO HEK293T cells transfected with the control plasmid (Ctrl), LRP5, CD36, or FATP2 were cultured in the charcoal-filtered medium overnight, followed by incubation with EPA for three hours. The co-localization of EPA and LAMP1 was detected using PLA (magenta signal). The quantification of the PLA signal is shown in (**h**) (n=13 for Ctrl, n=13 for LRP5, n=11 for CD36 and n=13 for FATP2). Data are presented as mean±sem with p values (One-way ANOVA). Bone marrow WT and LRP5 KO neutrophils were incubated with Celecoxib (**e**), NDGA (**f**) or Miconazole (**G**) for three hours in the presence of GM-CSF and analyzed by Western Blotting.

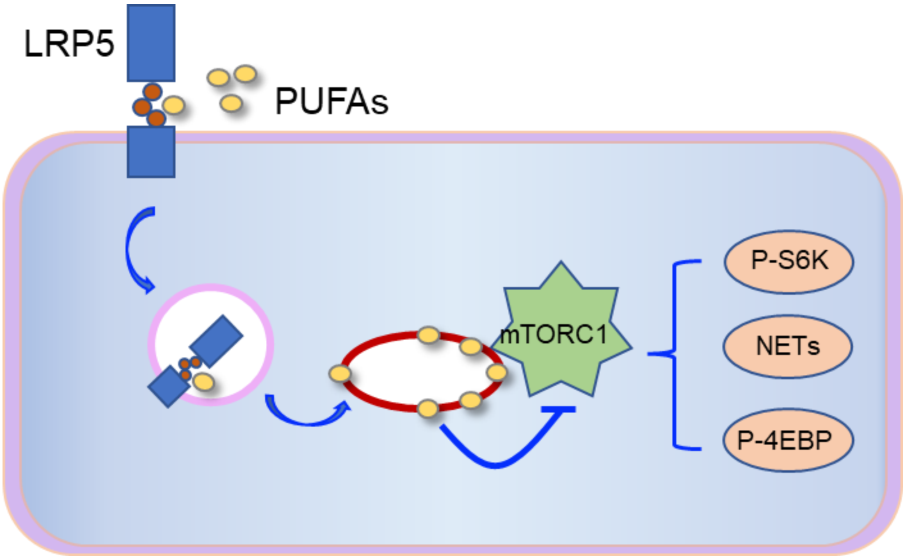

Supplementary Figure 7. Schematic representation of LRP5, through its LDLa domain, transports PUFAs to intracellular compartments including lysosomes, where it suppresses mTORC1 signaling to regulate cell functions.
