# Supplementary material for: LDL receptor-related protein 5 selectively transports unesterified polyunsaturated fatty acids to intracellular compartments
Source: Nat Commun. 2024 Apr 9;15:3068. doi: 10.1038/s41467-024-47262-z (PMC11004178; doi:10.1038/s41467-024-47262-z)
Supplement: Supplementary file 3 — Description of Additional Supplementary Files [file 41467_2024_47262_MOESM3_ESM.pdf]

**Description of Additional Supplementary Files**

**File Name: Supplementary Data 1**
**Description:** Lipidomic analysis raw data of WT and LRP5-KO neutrophils from mice on normal diet (p value was calculated using Student's t-test, Two-tailed, unpaired).

**File Name: Supplementary Data 2**
**Description:** Lipidomic analysis raw data of WT and LRP5-KO neutrophils from mice on EFA-Free diet
