# Supplementary material for: LDL receptor-related protein 5 selectively transports unesterified polyunsaturated fatty acids to intracellular compartments
Source: Nat Commun. 2024 Apr 9;15:3068. doi: 10.1038/s41467-024-47262-z (PMC11004178; doi:10.1038/s41467-024-47262-z)
Supplement: Supplementary file 6 — Reporting Summary [file 41467_2024_47262_MOESM6_ESM.pdf]

nature portfolio

Corresponding author(s): Wenwen Tang & Dianqing Wu

Last updated by author(s): Mar 9, 2024

Reporting Summary

Nature Portfolio wishes to improve the reproducibility of the work that we publish. This form provides structure for consistency and transparency in reporting. For further information on Nature Portfolio policies, see our Editorial Policies and the Editorial Policy Checklist.

Statistics

For all statistical analyses, confirm that the following items are present in the figure legend, table legend, main text, or Methods section.

n/a	Confirmed	
☐	☒	The exact sample size (*n*) for each experimental group/condition, given as a discrete number and unit of measurement
☐	☒	A statement on whether measurements were taken from distinct samples or whether the same sample was measured repeatedly
☐	☒	The statistical test(s) used AND whether they are one- or two-sided<br>*Only common tests should be described solely by name; describe more complex techniques in the Methods section.*
☐	☒	A description of all covariates tested
☐	☒	A description of any assumptions or corrections, such as tests of normality and adjustment for multiple comparisons
☐	☒	A full description of the statistical parameters including central tendency (e.g. means) or other basic estimates (e.g. regression coefficient) AND variation (e.g. standard deviation) or associated estimates of uncertainty (e.g. confidence intervals)
☐	☒	For null hypothesis testing, the test statistic (e.g. *F*, *t*, *r*) with confidence intervals, effect sizes, degrees of freedom and *P* value noted<br>*Give P values as exact values whenever suitable.*
☒	☐	For Bayesian analysis, information on the choice of priors and Markov chain Monte Carlo settings
☒	☐	For hierarchical and complex designs, identification of the appropriate level for tests and full reporting of outcomes
☐	☒	Estimates of effect sizes (e.g. Cohen's *d*, Pearson's *r*), indicating how they were calculated

Our web collection on statistics for biologists contains articles on many of the points above.

Software and code

Policy information about availability of computer code

Data collection	Immunofluorescence images were acquired using Leica Application Suite X (LAS X) software on Leica SP5 confocal. 14C-fatty acid Uptake signal were acquired using a liquid scintillation counter (PerkinElmer, Tri-CARB 2100TR). Flow imaging were performed using Amnis ImageStream-X MarkII Imaging. Western blot data were imaged using Image Lab software (Bio-Rad). Isothermal titration calorimetry data were collected using ITCRun software on Nano ITC TA instrument.
Data analysis	Statistical analyses were performed using GraphPad Prism 10.2.0. GraphPad Prism 10.2.0 was also used for plotting Volcano plots and Heat maps. Image J was used for biochemical analysis of Western blots and image analysis, including the Pearson's coefficients calculation for colocalization, of confocal images. IDEAS 6.2 was used for analyzing flow imaging data. RNA-seq was analyzed by running the DESeq2 pipeline (default parameters) and signaling pathway analysis was performed in IPA (Ingenuity Pathway Analysis). Flowcytometry analysis was done with Flowjo v10.8. No custom software was used.

For manuscripts utilizing custom algorithms or software that are central to the research but not yet described in published literature, software must be made available to editors and reviewers. We strongly encourage code deposition in a community repository (e.g. GitHub). See the Nature Portfolio guidelines for submitting code & software for further information.

Data

Policy information about availability of data

All manuscripts must include a data availability statement. This statement should provide the following information, where applicable:

- Accession codes, unique identifiers, or web links for publicly available datasets
- A description of any restrictions on data availability
- For clinical datasets or third party data, please ensure that the statement adheres to our policy

The RNAseq data has been deposited to Gene Expression Omnibus. The accession number is GSE195678. (To review the dataset please go to https://nam12.safelinks.protection.outlook.com/?url=https%3A%2F%2Fwww.ncbi.nlm.nih.gov%2Fgeo%2Fquery%2Facc.cgi%3Facc%3DGSE195678&data=05%7C02%7Cwenwen.tang%40yale.edu%7C46bae33c6700454d80a208dc11e6c136%7Cdd8cbebb21394df8b4114e3e87abeb5c%7C0%7C0%7C638404929993123803%7CUnknown%7CTWFpbGZsb3d8eyJWIjoiMC4wLjAwMDAiLCJQIjoiV2luMzIiLCJBTiI6Ik1haWwiLCJXVCI6Mn0%3D%7C3000%7C%7C%7C&sdata=zn63l4tfi2EUthPpBZvALx6P0TR%2FYGmLj1k%2By0TU4X4%3D&reserved=0; Enter token afqbuwiktxmzpqt into the box)

Source Data are provided with this paper.

Human research participants

Policy information about studies involving human research participants and Sex and Gender in Research.

Reporting on sex and gender	N/A
Population characteristics	N/A
Recruitment	N/A
Ethics oversight	N/A

Note that full information on the approval of the study protocol must also be provided in the manuscript.

Field-specific reporting

Please select the one below that is the best fit for your research. If you are not sure, read the appropriate sections before making your selection.

[x] Life sciences [ ] Behavioural & social sciences [ ] Ecological, evolutionary & environmental sciences

For a reference copy of the document with all sections, see nature.com/documents/nr-reporting-summary-flat.pdf

Life sciences study design

All studies must disclose on these points even when the disclosure is negative.

Sample size	Minimal group sizes for mouse studies were determined by using power calculations with the DSS Researcher's Toolkit with an α of 0.05 and power of 0.8.
Data exclusions	No data were excluded from analysis.
Replication	Animal experiments were repeated at least twice and all of in vitro experiments were repeated at least three times.
Randomization	All mice were randomly assigned to experimental groups. Randomization was done in other experiments when possible.
Blinding	Animals were grouped based on their genotype, so it was unblinded, but investigators were blinded for most of the quantification experiments. For other experiments, samples were unblinded after data collections have been completed.

Reporting for specific materials, systems and methods

We require information from authors about some types of materials, experimental systems and methods used in many studies. Here, indicate whether each material, system or method listed is relevant to your study. If you are not sure if a list item applies to your research, read the appropriate section before selecting a response.

Materials & experimental systems

n/a	Involved in the study
☐	☒ Antibodies
☐	☒ Eukaryotic cell lines
☒	☐ Palaeontology and archaeology
☐	☒ Animals and other organisms
☒	☐ Clinical data
☒	☐ Dual use research of concern

Methods

n/a	Involved in the study
☒	☐ ChIP-seq
☐	☒ Flow cytometry
☒	☐ MRI-based neuroimaging

Antibodies

Antibodies used	Citrullinated-Histone-H3 (Abcam, ab5103), Myeloperoxidase/MPO (R&D, AF3667), LAMP-1 (Santa Cruz, sc-20011), TGN38 (Santa Cruz, Sc27680), PUFA (Cloud-Clone, PAO623Ge01), Beta-Catenin (BD, 610154), p-AKT473 (Cell Signaling Technology, 4060), mTOR (Cell Signaling, 2983), LAMP1 (Santa Cruz, sc-19992), PerCP-Cy5.5-Ly6G (BD, 560602), Pacific blue-CD11b (Biolegend 101223), Lrp5 (Cell Signaling Technology, 5731), Lrp6 (Cell Signaling Technology, 3395), Phospho-S6 (Cell Signaling Technology, 4858), total S6 (Cell Signaling Technology, 2217), Phospho-p70 S6K (Cell Signaling Technology, 9234), total p70 S6K (Cell Signaling Technology, 2708), Phospho-4E-BP1 (Cell Signaling Technology, 2855), total 4E-BP1 (Cell Signaling Technology, 9452), Beta-Actin (Proteintech, 66009), and HSP90 (Proteintech, 13171). All flow cytometry antibodies were used with 1:100 dilution. All western antibodies were used with 1:1000 dilution.
Validation	Anti-PUFA antibody (Cloud-Clone, PA0623Ge01) was thoroughly validated in the manuscript (see manuscript for the details). No other antibody validation was performed in this study. All of these validated commercial antibodies used in the study were chosen based on manufacturer's validation results or references on their websites.

Eukaryotic cell lines

Policy information about cell lines and Sex and Gender in Research

Cell line source(s)	HEK293T (ATCC: CRL-3216), Expi293F™ Cells (Thermo Fisher, A14527)
Authentication	Cells were authenticated by the source and checked in lab for appropriate morphology.
Mycoplasma contamination	Cells have been routinely tested for mycoplasma and they were negative.
Commonly misidentified lines (See ICLAC register)	None were used.

Animals and other research organisms

Policy information about studies involving animals; ARRIVE guidelines recommended for reporting animal research, and Sex and Gender in Research

Laboratory animals	The LoxP-floxed Lrp5 (Lrp5fl/fl) and Lrp6 (Lrp6fl/fl) mice were obtained from Bart Williams. The Lrp5fl/fl and Lrp6fl/fl mice were back-crossed with C57BL/6N mice for more than seven generations before being intercrossed with Lyz2-Cre (#004781), Rosa26-CreER2 (#008463), or Mrp8-Cre (#021614) mice (Jackson lab), followed by additional backcrossing with C57BL/6N mice for more than ten generations. Ncr-1-Cre Lrp5fl/fl mice were described previously. Wildtype C57BL/6N mice were purchased from Envigo (#044). The mice were housed under specific-pathogen-free conditions in Yale Animal Resources Center facilities and under 12 hr light/dark cycles at 68-79°F and 30-70% humidity.
Wild animals	No wild animals were used.
Reporting on sex	The findings apply to both sexes.
Field-collected samples	No field-collected samples were used.
Ethics oversight	All animal experiments were performed with the approval of Institutional Animal Care and Use Committee (IACUC) at Yale University.

Note that full information on the approval of the study protocol must also be provided in the manuscript.

Flow Cytometry

Plots

Confirm that:

- [x] The axis labels state the marker and fluorochrome used (e.g. CD4-FITC).
- [ ] The axis scales are clearly visible. Include numbers along axes only for bottom left plot of group (a 'group' is an analysis of identical markers).
- [x] All plots are contour plots with outliers or pseudocolor plots.
- [x] A numerical value for number of cells or percentage (with statistics) is provided.

Methodology

Sample preparation	Analysis were performed on dissociated single type cells. Blood cells were collected and fixed with 2% PFA (Santa-Cruz sc-281692) after lysing red blood cells. Hearts were minced using scissors and scalpel blades and incubated with a digestion buffer [RPMI1640, 5% FBS, 1% PS, 25mM HEPES and 300U/ml collagenase(Sigma C0130)] in a shaker for 20-25min at 37°C. Disperse cells were filtered through a 70µm cell strainer to eliminate clumps and debris. After centrifugation for 5 minutes (500xg) at 4°C, cell pellets were resuspended in the Red Blood Cell Lysis Buffer (Sigma R7757) and incubated at RT for 5 min to remove erythrocytes. Cells were pelleted again, resuspended and incubated with 1µg/ml Type I DNase (Sigma D4263) in RPMI1640 with 5% FBS for 5 min to digest DNA. Cells were then filtered again by a 40 µm cell strainer. Finally, the cells were pelleted and fixed with 2% PFA.
Instrument	BD LSRII flow cytometer
Software	Flowjo v10
Cell population abundance	N/A
Gating strategy	Cells were identified with FSC/SSC gates, debris and doublets were excluded, and fluorescent cells were identified based on non-fluorescent cell controls. Cells were gated with various markers as indicated in the figures.

- [x] Tick this box to confirm that a figure exemplifying the gating strategy is provided in the Supplementary Information.
